# Evaluation of the penetration of CHX 2% on dentinal tubules using Conventional Irrigation, Sonic Irrigation (EDDY) and Passive Ultrasonic Irrigation (PUI) techniques: An in vitro study

**DOI:** 10.4317/jced.57065

**Published:** 2021-01-01

**Authors:** Hair Salas, Andrés Castrejon, Dante Fuentes, Alexandra Luque, Edson Luque

**Affiliations:** 1 DDs, MSc, PhD. Facultad de Odontología de Universidad Católica de Santa María, Arequipa, Perú; 2 DDS. Práctica privada exclusiva a la endodoncia

## Abstract

**Background:**

Various procedures and techniques have been developed to increase the effectiveness and penetration of irrigants into the dentinal tubules and anatomical anfractuosities. Objective: The purpose of this in vitro study was to compare the effect of different irrigation techniques such as conventional irrigation with a syringe, sonic activation irrigation with EDDY and Passive Ultrasonic Irrigation. All these on dentinal tubule penetration using Chlorhexidine.

**Material and Methods:**

45 lower premolar teeth extracted for orthodontic reasons were used. These teeth were decoronated to a length of 18mm. This working length was achieved by inserting a size 15 K file into the root canal until it was observed in the ápex. Then the length was reduced to 1 mm. The instrumentation was performed with the Wave One Gold system up to a file # 45. Irrigated with 2mL of CHX between instruments. A final irrigation was performed using 5 mL of 17% EDTA with an E1 - Irrisonic insert for 30 seconds. Then, the root canal was irrigated with 5 mL of distilled water and dried with paper tips. The final irrigation of the 2% CHX with Rhodamine B was carried out with the different techniques of irrigation. Syringe irrigation with 5 mL, sonic irrigation and passive ultrasonic activation activated for 30 seconds in two stages. Axial cuts were made at 200 microns, the observation was done with stereomicroscope and image analysis in Image J software.

**Results:**

Statistically significant differences were found only in the apical region, where the depth penetration in the PUI group was 76 µm, MI 48 µm and SI 41 µm, while in the penetration area, the PUI group was 99 µm2, MI 77 µm2 and finally SI 53 µm2.

**Conclusions:**

The CHX was able to penetrate the dentinal tubules of three-thirds of the roots, for which the technique that showed the greatest penetration capacity was the ultrasonic activation.

** Key words:**Clorhexidine. Dentinal tubule. Dentinal penetration. Passive ultrasonic irrigation. Eddy. Manual irrigation.

## Introduction

The primary goals of root canal treatment are to eliminate pulp tissue, mineral debris, organic debris, microorganisms and their by-products from the root canal system (1). through chemomechanical preparation, the necrotic tissue, bacteria and their products are eliminated from the root canal (2).

However, this goal is not easy to achieve because even in the main canal, instruments have been shown not to touch all the walls of the canal. This happens because of factors related to instrument features and/or canal anatomy (2).

The microorganisms can penetrate areas that are difficult to clean mechanically such as in isthmus, ramifications and lateral or accessory canals, apical deltas and dentinal tubules (3).

Bacterial penetration to about 300 µm deep into the dentinal tubules has been reported (4) especially *Enterococcus faecalis* can extend 500µm deeply into human dentin (5). Endotoxins from gram-negative bacteria can penetrate approximately 300–500µm into dentinal tubules too (6). This infected or contaminated dentin might serve as a potential source for persistent apical periodontitis (7).

The use of irrigants is important for the removal of bacteria and organic tissue remnants from uninstrumented surfaces (8).

Sodium hypochlorite (NaOCl) and chlorhexidine digluconate (CHX) are the widely used root canal irrigants in endodontic therapy (9).

Recently Zandi, *et al.* (10) have shown that CHX can be used as a main irrigant, obtaining outcomes similar to those obtained with NaOCL, even in spite of its inability to dissolve organic tissue and biofilm (11).

CHX attacks the microbial cell wall or outer membrane resulting in the killing of the microbe (12).

Syringe irrigation remains is widely used as demonstrated by a survey conducted among the members of the AAE (13).

To increase the penetration and effectiveness of irrigants into the dentinal tubules and anatomical anfractuosities, various procedures and techniques to facilitate the penetration of irrigants have been developed (14).

There is a new sonic irrigation activation system called EDDY (VDW, Munich, Germany). It is a sterile single-use instrument non-cutting, made of flexible polyamide with a size of 25.04. This must be activated with 5000 to 6000 Hz by an air-driven handpiece (Air Scaler) (15).

According to the manufacturer, the instrument is claimed to create a three-dimensional movement that triggers “cavitation” and “acoustic streaming”. This allows efficient cleaning of complex root canal systems without the limitations of ultrasound (15).

Another kind of irrigation activation system is the use of passive ultrasonic irrigation (PUI). In this technique, the irrigant is agitated through two physical effects, acoustic microstreaming and cavitation, its effects improve the action of irrigants.

Several studies have shown that PUI irrigation improves the cleaning of complex anatomic areas in the canal (16).

Ackay, *et al.* (17) has shown the effect of PUI on the penetration of dentinal tubules after use sodium hypochlorite, but there is no consensus (18).

Regarding the penetration depth of irrigants into root dentinal tubules, there is some information available. However, there are few studies específically about CHX (8,9,19).

The purpose of this *in vitro* study was to compare the effect of different irrigation techniques such as conventional irrigation with a syringe, sonic activation irrigation with EDDY and PUI irrigation on dentinal tubule penetration using CHX.

## Material and Methods

A total of 45 human mandibular premolar teeth, freshly extracted for orthodontic reasons, were used for this study. The Ethical Committee of the Santa María Catholic University (Arequipa, Perú) approved the study. The selected teeth had straight and round root canals. They were stored in 0.1% thymol solution at 5 ° C for no more than 1 month.

The samples were distributed in 3 groups of 15 teeth each and were categorized by the different systems used: Manual Irrigation (MI group), Sonic Irrigation (SI group) and Passive Ultrasonic Irrigation (PUI group).

Samples were cut using a diamond disk to obtain a standardized root length of 18 mm to eliminate coronal interference and obtain a flat reference for instrumentation.

The working length was made by inserting a size 15 K-file (Dentsply, Maillefer, Ballaigues, Switzerland) into the root canal until it could be observed at the foramen, then the length was reduced 1 mm. Root canals of each tooth were shaped using Wave One Gold system (Dentsply, Switzerland) up to a 45 file in a crown down manner.

The samples were irrigated with 2 ml of 2% CHX solution (Maquira, Maringa, Brazil) between each instrument. After washed was performed using 17% EDTA (Maquira, Maringa, Brazil) with an E1 - Irrisonic insert (Helse Ultrasonic, São Paulo, Brazil). It was placed until 1 mm of the working length and the ultrasonic device (P5 Newtron XS, Satelec Acteon Group, Merignac, France) was activated for 30 sec at 25% of intensity. Finally, the root canal was irrigated with 5 mL of distilled water and dried with paper cones.

-Final irrigation procedure

Approximately 0.1 g of rhodamine B dye (Sigma Aldrich, Bengaluru, India) was mixed with 500 ml of 2% CHX solution to provide visualization within the dentinal tubules.

MI group: In this technique, a needle NaviTip (30 ga, Ultradent, Köln, Germany) with manual up-and-down movement of the needle inside the canal, was placed into the root canal until 1 mm of the working length. 5 mL of CHX 2% + Rhodamine B were used.

SI group: In this technique, 0.5 mL of 2% CHX + Rhodamine B was placed in the root canal; an EDDY tip (polyamide tip, VDW, Munich, Germany) was inserted into the root canal up to 1 mm of working length. Then, it was activated for 30 seconds in short vertical strokes of 2–3 mm. 2 times.

PUI group: In this technique, 0.5 mL of CHX 2% + Rhodamine B was placed into the canal. An E1 - Irrisonic insert (Helse Ultrasonic, São Paulo, Brazil) was used, it was placed until 1 mm of the working length with an up-and-down motion. Then, the ultrasonic device (P5 Newtron XS, France) was activated similar to when the smear layer was removed. It was made 2 times.

Teeth were embedded in methyl methacrylate resin (Technovit 3040; Heraeus Kulzer GmbH & Co. Wehrheim, Germany), and transverse sections of 200µm thickness were obtained perpendicularly to their long axis in the apical, middle and coronal thirds of each tooth using a diamond disk adapted in a low-speed cutting machine (Isomet 1000, São Paulo, SP, Brazil). Under continuous water irrigation.

Samples were examined with stereomicroscopy (Leica DM750, Mannheim, Germany) with an increase of X4. The images were first calibrated in the microscope software. The average penetration depth was measured using the straight-line tool of the Image J software (National Institutes of Health, Bethesda, MD, USA), in 8 standardized points. This was done for all cuts.

With the Image J polygon selection tool the average penetration area was evaluated by measuring the originally stained portion in the root canal and subtracting the circumference value of the root canal. All specimens were evaluated by a single operator blinded to the groups.

-Statistical analysis

The sample size is estimated using the formula for finite but unknown populations; we established a confidence value of 95% (1.96), probability of the phenomenon occurring of 99% and probability of failure of 1%, and the sampling error of 5%. 15 samples were required for each group.

We use the Shapiro - Wilk normality test to evaluate the normal distribution. The statistical analysis for all the parametric data was the ANOVA test (all), and complementing this statistical analysis was the TUKEY test for the significant difference.

## Results

**Figure 1:**
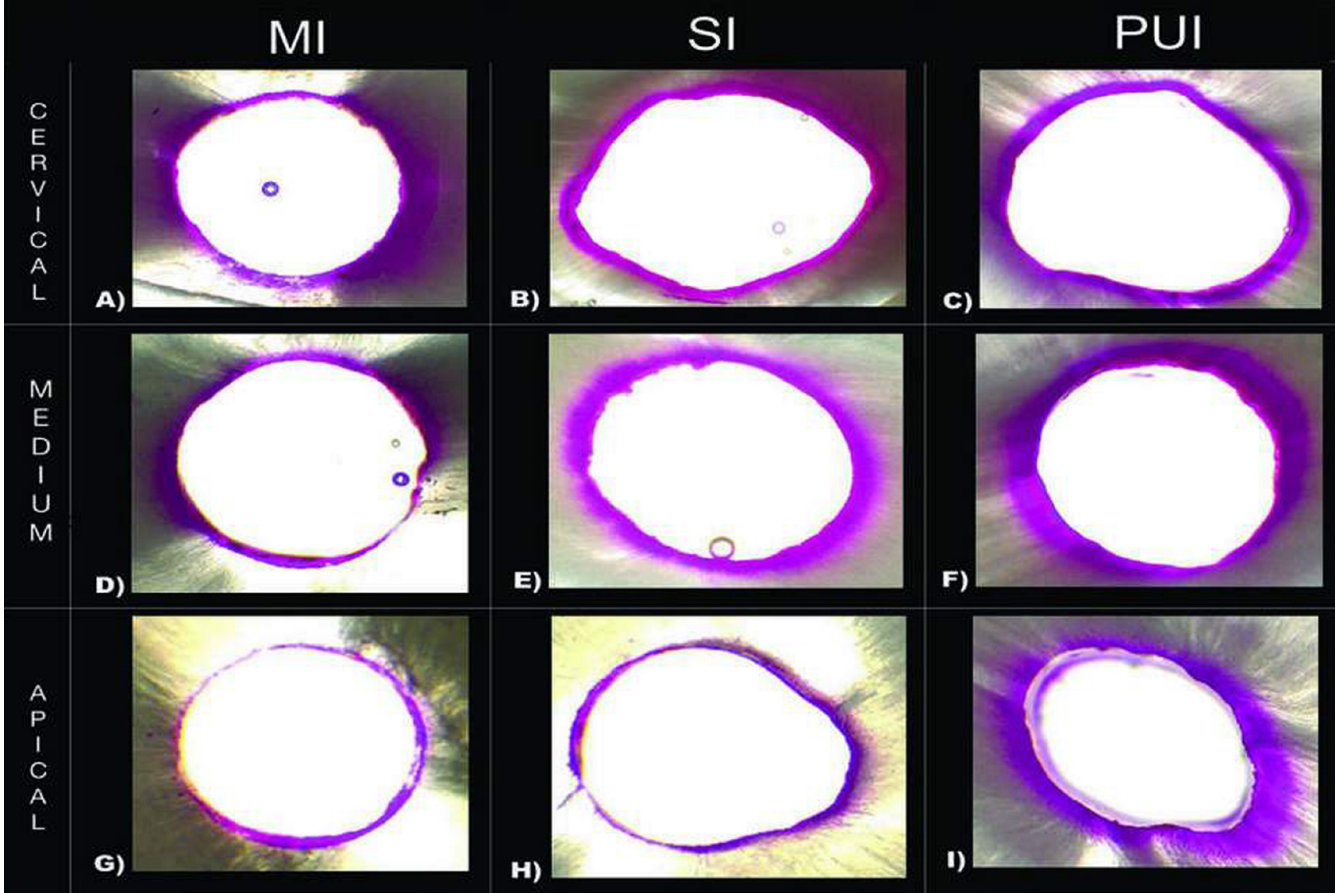
Different irrigation methods at different levels: A) Manual Irrigation Cervical B) Sonic Irrigation Cervical C) Passive Ultrasonic Irrigation Cervical D) Manual Irrigation Medium E) Sonic Irrigation Medium F) Passive Ultrasonic Irrigation Medium G) Manual Irrigation Apical H) Sonic Irrigation Apical I) Passive Ultrasonic Irrigation Apical.

**Figure 2:**
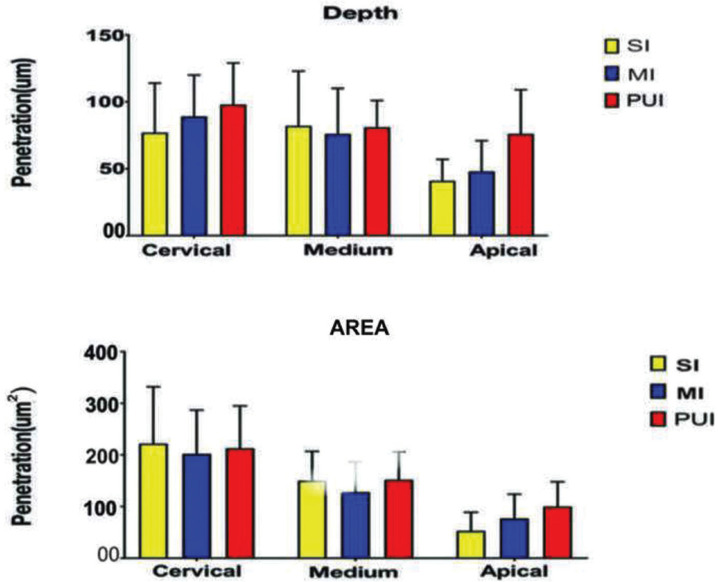
Area and depth penetration of chlorhexidine into dentinal tubules, the best activation technique was PUI. The penetration depth in PUI (76µm) and the penetration area was PUI (99µm2).

Three irrigation methods were tested, which were evaluated in the cervical, middle and apical regions (Fig. 1). These were tested at depth level and area level. Statistically significant differences were found only in the apical region, where the mean PUI group was greater than the SI group; no differences were found between the other comparisons, one-way ANOVA, *P* > 0.05 (Fig. 2).

In the evaluation of depth level in the cervical area it was given as follows SI (77µm) <MI (89µm) <PUI (98µm). In the middle area the penetration depth was given as follows MI (76µm) <PUI (81µm) <SI (82µm). In none of the previous two, there were significant differences (Table 1). However, in the apical area there was (*p* <0.05), given in the following order: SI (41µm) <MI (48µm) <PUI (76µm). In the separate evaluation of the depth in the apical area, it was found that PUI had greater depth than SI and MI (*p* < 0.05).

**Table 1:**
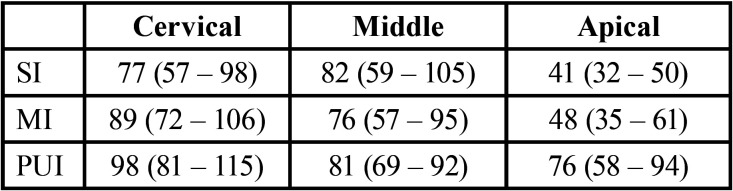
Depth Penetration (µm). In depth penetration, evaluation was found that in cervical region PUI had better results than MI and SI. In the middle region, SI had better results than PUI and MI. Finally, in apical region PUI had way far better results than MI and SI.

In the evaluation of the area, in the cervical third was found that MI (202µm2) < PUI (213µm) <SI (222µm2). In the middle area the distribution was MI (128µm2) < SI (149µm2) < PUI (152µm2), finding only statistically significant differences (*p* <0.05) in the apical area: SI (53µm2) = MI (77µm2) and MI (77µm2) = PUI (99µm2), but SI (53µm2) < PUI (99µm2) (Table 2).

**Table 2:**
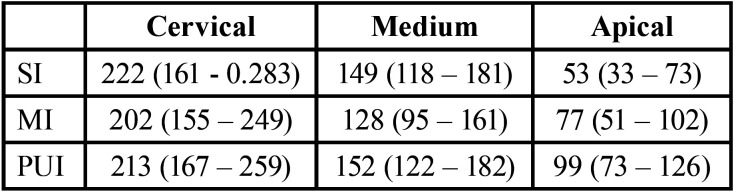
Area Penetration (µm2). In area penetration evaluation in the cervical region it was found that MI <PUI <SI. In the middle zone the distribution was MI <SI <PUI. Finding only statistically significant difference. In the apical zone: SI < MI <PUI.

## Discussion

Endodontic therapy consists of eliminating the greatest amount of bacteria that have not only invaded the root canal but also the dentinal tubules, of course, this is effectively achieved by careful instrumentation and irrigation.

Various irrigants such as chlorhexidine, sodium hypochlorite, tetraclean, green tea polyphenols, and recently a new irrigant Octenidine Dihydrochlorideson have been tried to help in the disinfection. However, NaOCL is the most used during the irrigation (20).

On the other hand, CHX is often used as a final irrigant after EDTA (11). However, recently has been reported that CHX, when is used as main irrigant, has similar success rates to those obtained with NaOCL (10). This is probably because the CHX is a broad-spectrum synthetic cationic bis-guanide antimicrobial agent, which also presents an additional benefit that is its adsorption to the dentine and slow-release posterior due to a phenomenon known as substantivity that extends its antimicrobial activity (21).

It has been suggested that CHX not only bounds to mineralized dentine but also to demineralized dentin (21).

On the demineralized dentine, CHX can diffuse through the collagen matrix and remain trapped within the spaces between the collagen fibrils (22).

However, few studies have evaluated the penetration depth of CHX in dentinal tubules. Most studies had evaluated the penetration of NaOCL into the tubules using the technique of bleaching of dye technique. In the meantime, this technique cannot be used with CHX because CHX has no bleaching properties.

In previous studies, rhodamine B dye was added with CHX to evaluate their penetration depth into dentinal tubules, since by mixing CHX with rhodamine B the surface tension of CHX could be altered. In our study, just like Vadhana, *et al.* (9) the surface tension of 2% CHX and 2% CHX mixed with rhodamine B dye were evaluated previously, the results confirmed to be similar, this was probably due to the small quantity of the dye mixed with the irrigant.

Many factors can influence the penetration depth of irrigant into dentinal tubules. There are proper factors of the tooth. For example, the intricate anatomy of the root canal system, it also must be considered that there are more dentinal tubules with a larger diameter in the coronal area than the apical zone (23).

On the other hand are the factors related to the irrigating solution such as, viscosity and surface tension which are two main factors that influence the flow and penetration depth of the irrigants (24,25).

In our investigation in all experimental groups, the main penetration was observed at the cervical third and the lower in apical third, which agrees with previous studies (8,9).

Another factor that influences the deeper penetration of CHX into dentinal tubules is the remotion of the smear layer. The use of quelators like EDTA had demonstrated to be effective in dentinal tubule opening, this facilitates the penetration of the irrigating solution (26). we agree with this.

Irrigation and especially activation of irrigation solutions are crucial for further improvement of canal cleanliness and disinfection of the entire root canal system (16).

In the present study, the area and depth of penetration of the irrigant within the tubules of the canal wall did not give significant differences for the 3 groups at the cervical and middle area level. However, statistically significant differences were found only in the apical third where the PUI group was better. In general terms, the depth of penetration decreased from the coronal to the apical third of the root canals. These results are in agreement with previous studies (9,20).

During syringe Irrigation, a steady laminar flow is developed (27). This flow is not necessarily turbulent.

This laminar flow during irrigation will create low frictional forces (wall shear stress) between the flowing irrigant and the root canal walls (28).

We think that even though during syringe irrigation the flow is laminar and the wall shear stress is minimal, the mechanical effect of the irrigant is enough to allow penetration into the dentinal tubules to be similar on both activation systems.

No significant differences were present between both activation systems. However, more efficient penetration of CHX into dentinal tubules was with passive ultrasonic irrigation (PUI).

The best results obtained during PUI can be explained because of some physical effects that occur. The first physical effects can be defined as acoustic streaming. It is a rapid movement of the fluid in a circular or vortex shape around the vibrating file which can attract debris and bacteria from the wall to the vortex (29). Another physical effect is the cavitation which is the formation, behavior, and collapse of bubbles. The collapse of these bubbles close to a wall can generate a high-velocity jet directed towards the wall releasing the smear layer and thus enhancing its cleaning (30).

We believe that the high-velocity jet directed towards the wall not only enhances its cleaning but also forces the irrigating solution to penetrate the tubules.

Van der Sluis, *et al.* (29) shown a rise of the intracanal temperature when the irrigant was ultrasonically activated for 30 seconds without replenishment.

We think that the increase in CHX temperature during PUI may have helped to reduce its surface tension and therefore have contributed to improving the penetration capacity of the irrigator within the tubules.

To our knowledge, no studies are assessing the depth of penetration of CHX into dentinal tubules using Eddy.

Penetration of CHX into dentinal tubules was more efficient with SI compared to MI group. This can be explained by the fact that EDDY works with 6000 Hz. It will generate cavitation and acoustic transmission, phenomenons to which can be recognized as the best results.

In the meantime, better results were obtained when the PUI group was compared with the SI group; although the differences were not significant, our results agree with other studies that have shown that the use of an ultrasonic irrigation exhibited significantly more penetration than sonic irrigation (17).

## Conclusions

The three irrigation methods that were tested showed similar depth and area of penetration of CHX into dentinal tubules in the cervical and middle level. Statistically significant differences were found only in the apical region, where the mean PUI group was greater than the SI group. No differences were found between the other comparisons.
